# A comparative evaluation of the effect of addition of 8 mg dexamethasone to 2% lignocaine with adrenaline in mandibular third molar surgery: a split mouth randomised double blind study

**DOI:** 10.3389/froh.2024.1349832

**Published:** 2024-02-09

**Authors:** P. Poorna, Premalatha Shetty, Veerbhadra Kalyani, Sameep Shetty, Madhusudan Upadya, Prasanna Mithra

**Affiliations:** ^1^ Department of Oral and Maxillofacial Surgery, Manipal College of Dental Sciences Mangalore, Manipal Academy of Higher Education, Manipal, India; ^2^ Department of Oral Oncosurgery, VTSM Peripheral Cancer Centre, Kalaburagi, India; ^3^ Department of Emergency Medicine, Kasturba Medical College, Mangalore, Manipal Academy of Higher Education, Manipal, India; ^4^ Department of Community Medicine, Kasturba Medical College, Mangalore, Manipal Academy of Higher Education, Manipal, India

**Keywords:** impacted tooth, third molar, local anaesthesia, dexamethasone, steroids

## Abstract

**Background:**

Impacted lower third molar surgeries involve trauma in a highly vascularized zone with loose connective tissue leading to inflammatory sequelae including postoperative pain, swelling, trismus and generalised oral dysfunction during the post-operative phase. In minor oral surgical procedures, an all-inclusive method to protract anaesthesia and reduce the inevitable post-operative sequelae is yet to be explored substantially.

**Aim:**

To evaluate the efficacy of dexamethasone added to local anaesthetics in extending the depth and duration of anaesthesia and decreasing the postoperative complications after surgical removal of impacted third molars.

**Methodology:**

A controlled, randomized, split-mouth, double-blind prospective study involving lower third molar surgery was performed in 35 patients wherein the test group (Group I) received 8 mg dexamethasone added to 2 ml of 2% lignocaine with epinephrine and the control group (Group II) received 2 ml of sterile water added to 2 ml of 2% lignocaine with epinephrine. Onset and duration of anaesthesia were evaluated; followed by evaluation of pain, swelling and trismus for 7 days post-surgery, using independent *t*-test and ANOVA for repeated measures.

**Results:**

Test group had a faster onset of anaesthesia by 69 s and a lengthier duration of 128.4 min (*p* < 0.001). Pain scores (Visual Analogue Scale) in the first 24 h were 4.9 and 7.5 in the test and control group respectively (*p* < 0.001). The average dosing of analgesics until postoperative day 7 in the test and control group were 12.6 and 18.4 respectively (*p* < 0.001). The swelling was significantly lesser in the test group, in addition, trismus was significantly lesser by 1 cm on postoperative days 1 and 2 and 0.2 cm on day 7.

**Conclusion:**

The addition of dexamethasone to lignocaine in the nerve block reduces the time of onset and significantly prolongs the duration of anaesthesia with decreased pain, swelling and trismus. Steroids mixed directly with the local anaesthetic agent can minimise the post-operative sequelae associated with third molar surgery with a single needle prick.

## Introduction

Surgical extraction of impacted third molars is a routine minor oral surgical procedure carried out under local anaesthesia. Many approaches have been tried to smoothen the post-operative phase after surgical extraction of impacted third molars, including pharmacological and nonpharmacological measures (1). Without sufficient depth and duration of local anaesthesia minor surgical procedures bring in a comparative amount of pain and discomfort (2).

Surgical removal of third molars is one of the most common procedures in oral surgery resulting in pain, swelling, bleeding, infection, trismus, and paraesthesia that may be transitory or permanent (3–5). The post-operative sequelae are elicited by an inflammatory response, leading to vasodilation and, an influx of pro-inflammatory mediators: histamine, bradykinin, and prostaglandins (6–8).

Studies done in other disciplines of surgery (9–12) and *in vivo* studies have shown that using corticosteroids as an adjunct to the local anaesthetic agent has increased its duration of action.

Perineural dexamethasone, as an adjuvant to peripheral nerve block, has been associated with a faster onset of anaesthesia, longer duration of anaesthesia/analgesia, reduction in postoperative pain intensity and analgesia requirements compared with the use of local anaesthetic alone (13–16).

The reduction in pain intensity and the protracted analgesia attained with the use of dexamethasone as an adjunct to local anaesthesia may be due to these plausible factors. (a) Dexamethasone acts on glucocorticosteroid receptors to induce vasoconstriction, thereby minimising the pooling of local anaesthetic into the systemic circulation (16). (b) The inhibition of C-fibre transmission of pain signals and its direct action on the nerve cell to diminish neural discharge (17, 18).

Sizeable research in similar lines of coalescing local anaesthetic agents with dexamethasone has not been done in the field of Oral and Maxillofacial Surgery (19–22).

Lignocaine an amide local anaesthetic is a boon for surgeons to perform minor surgical procedures at ease obviating the need for general anaesthesia. Lignocaine when mixed with dexamethasone forms a drug combination that deserves testing (23). Studies done previously have shown that the resultant solution after mixing dexamethasone with lignocaine is chemically stable and has a higher pH, refining the patient experience at the time of administration of local anaesthesia while reducing the time of onset and increasing the duration of action of the local anaesthetic agent (24).

Dexamethasone is a synthetic glucocorticosteroid, devoid of mineralocorticoid effect (25).

It inhibits vascular dilation, and fluid transudation, and has a marginal unfavourable influence on leukocyte chemotaxis all of which contribute to post-operative swelling and trismus (26). It is 25–50 times more potent than hydrocortisone, has a plasma half-life of 100–300 min, a biological half-life of 36–72 h and is considered one of the most potent anti-inflammatory drugs (27).

At an inflammatory dose, dexamethasone lacks the sodium-maintaining properties of hydrocortisone. Further, it also controls the rate of synthesis of anti-inflammatory genes (28–30).

A 4 mg dose can produce five times the body's standard physiological output of cortisol (31). The onset of dexamethasone is assumed to be 1–2 h—sufficient time to diffuse along the cell membrane (32). Corticosteroids are efficacious during the first 24 h after surgery and continue to display their effects for up to three days (26).

The study's primary outcome was to evaluate the efficacy of dexamethasone as an adjunct to lignocaine with adrenaline in comparison with lignocaine with adrenaline alone in increasing the depth and duration of local anaesthesia. The secondary outcome was to evaluate the efficacy of the steroid local anaesthetic mixture in reducing the post-operative sequelae such as pain, swelling, and trismus and to report any incidence of adverse events following the administration of the twin mixture.

## Methods

This was a prospective, split-mouth, randomized, double-blinded study, carried out in Manipal College of Dental Sciences, Mangalore, Department of Oral and Maxillofacial Surgery among patients attending the outpatient department for removal of impacted mandibular third molars between December 2020 and November 2022. Sample size was calculated using the following formula as 70.

$$n\;=\;\frac{2{\left[Z_{1-\frac{\alpha }{2}}+Z_{1-\beta \;}\right]}^{2}\sigma^{2}}{d^{2}}$$

$Z_{1-\frac{\alpha }{2}}\;=\;1.96$ is a standard normal value at 5% level of significance.

$Z_{1-\beta \;}\;=\;0.84$ is a standard normal value at 80% power

σ = combined standard deviation = 2.195

d = clinically significant difference = 1.5

With 95% confidence interval, the sample size in each bilateral was 35, the total sample size was 70.

After Institutional Ethics Committee (IEC) approval, patients visiting the Oral and Maxillofacial Surgery outpatient department for surgical extraction of impacted mandibular third molars were screened. After obtaining written, informed patient consent, 35 ASA (American Society of Anaesthesiologists) physical status II patients aged 18–45 years requiring surgical extraction of bilateral mandibular third molars in class II position B, with no acute inflammation, excessive caries, pain and pathology around the mandibular third molars were included in the study. Subjects with active infection around impacted mandibular third molars; history of peptic ulcers, diabetes mellitus, systemic endocrine disorders, hypertension, renal diseases, bleeding diatheses, obesity, allergy to any of the drugs or materials used, use of antibiotics in past 2 weeks, use of NSAIDs in the past 1 week; pregnant and lactating mothers and those not willing to take part in the study were excluded from the study.

### Screening

Patients who visited the Department of Oral and Maxillofacial Surgery, Manipal College of Dental Sciences, Mangalore for surgical removal of impacted third molars were screened. During the first contact with the patient, possible candidature with bilateral impaction and gingival coverage enabling flap closure without tension was confirmed clinically and the position and relationship of the mandibular third molar to the overlying bone and adjacent tooth was confirmed radiologically by obtaining an Orthopantomogram or an Intraoral periapical radiograph. The existence of any possible reason for exclusion was assessed based on the clinical history and the radiographs obtained. Patients were provided with information regarding the surgical procedure and clinical study.

All the selected cases did not have any signs and symptoms of pain, trismus, or swelling at the time of surgical removal of impacted mandibular third molars.

Randomization and blinding procedure: After obtaining written informed consent and repeat confirmation that subjects have met the inclusion criteria, data regarding demographic and clinical variables were recorded. Demographic variables included name, age and sex (male or female) of the subject. Clinical variables included were the use of contraceptives (response: yes or no) in the last month, the use of psychotropic drugs (response: yes or no) and tobacco smoking (expressed as the number of cigarettes per day at the time of intervention). The recruited study subjects were then given a unique subject code each and were randomized based on the side (left or right), using simple randomization. The random numbers were generated using Microsoft EXCEL software. All odd numbers were allotted to the left side and even numbers to the right side, which got the intervention with 2 ml of 2% Lignocaine with 1:200,000 Adrenaline and 2 ml of 8 mg Dexamethasone, while the other side got 2% Lignocaine with 1:200,000 Adrenaline and 2 ml water for injection.

34 Allocation concealment was done using “opaque envelope method” where in each subject was assigned an opaque box containing the items to be injected in their respective containers with the side to be injected mentioned clearly on the label. The unique subject code of the subject was written over the opaque box.

Blinding - The administration was done by the operator independently, where the preloaded syringe was given to the operator by the co-investigator responsible for randomising and maintaining the opaque boxes. The 5 ml syringes containing 2 ml of 2% Lignocaine with 1:200,000 Adrenaline mixed with 2 ml of 8 mg Dexamethasone comprised the test group, while the control group comprised of 2 ml of 2% Lignocaine with 1:200,000 Adrenaline mixed with 2 ml water.

Each side of the subjects was either included in the test group (2 ml of 2% lignocaine with 1:200,000 adrenaline added to 2 ml of 8 mg dexamethasone) or the control group (2 ml of 2% lignocaine with 1:200,000 adrenaline added to 2 ml water for injection).

All subjects were operated by the same operator to minimise the differences due to operator variability. During the surgical process the mouth was rinsed for 20 s using 0.12% chlorhexidine mouthwash. 2 ml of 2% Lignocaine with 1:200,000 Adrenaline and 2 ml of 8 mg Dexamethasone, was loaded in a 5 ml syringe for the test group. 2 ml of 2% Lignocaine with 1:200,000 Adrenaline and 2 ml distilled water was also loaded in a 5 ml syringe for the control group. The inferior alveolar nerve, lingual nerve and long buccal nerve block were administered as per the randomization chart. The needle size used was 26 gauge 45 × 38 mm, 1.5 inch length.

Simple randomisation was done by random numbers generated using Microsoft EXCEL software. Both groups had 35 impaction sites and each subject was under his/her control. Allocation concealment was done using the “opaque envelope method”. The administration was done by the operator independently, wherein the pre-loaded syringe was given to the operator by the co-investigator responsible for randomising and maintaining the opaque envelopes.

Surgery was performed by the same surgeon using the same technique (buccal guttering and tooth sectioning) for all the patients. On the day of the surgical procedure, 1 g amoxicillin was given to all the subjects pre-operatively. A single dose of antibiotic covers the entire perioperative risk period and minimises the risk of antibiotic resistance and adverse effects. Repeat administration for prevention of infection is not necessary, further increasing patient compliance and reducing errors of administration. Ideally, an antibiotic should be administered 30 min before incision to achieve a stable tissue concentration. For surgical procedures lasting longer than 3 h, a second dosage is recommended, but not applicable to our study. A single dose of 1 gm amoxicillin was recommended as its plasma concentration is higher than the minimum inhibitory concentration needed to prevent the common bacteria involved in surgical infections (33–36).

Facial measurements were taken using a 2–0 nylon thread and a milli meter ruler before the surgery; 24 h, 48 h and 1 week after the surgery. The measurements were taken by marking the angle of the mandible, the tragus, the labial commissure, the nasal border, laterally to the external corner of the eye, and on the soft pogonion with a permanent marker. Distances measured were

D1 - Angle of the mandible to Tragus
D2 - Angle of the mandible to the external corner of the eye
D3 - Angle of the mandible to Nasal border
D4 - Angle of the mandible to Labial commissure
D5 - Angle of the mandible to soft tissue Pogonion.

Facial oedema is difficult to compute precisely as it involves 3 dimensional measurement of an irregular convex surface internally, as well as externally. The oedema and swelling associated with the surgical trauma can augment the associated trismus which has several contributing factors.

Mouth opening was assessed by measuring the inter-incisal distance using a divider pre-operatively, 24 h, 48 h and 1 week after the procedure.

All the measurements were recorded using a Proforma. Subjects were recalled after 4 weeks for surgical extraction of the third molar on the contralateral side following the same protocol.

4 ml of local anaesthetic agent mixed with either dexamethasone or sterile water for injection was administered to block inferior alveolar, lingual and long buccal nerves, following the blinding and randomisation protocols. Time taken for anaesthetic onset was recorded as the time from administration of the block to until the subject reported no pain on an atraumatic prick in the canine and molar region checked at 20-second intervals. Surgical extraction of the impacted mandibular third molar was done in a sterile environment under local anaesthesia. Duration of anaesthesia was recorded from the time when the individual experienced mild-moderate pain until the patient reported of no pain upon atraumatic prick. All subjects were prescribed Paracetamol 650 mg orally SOS and Chlorhexidine mouthwash 3 times a day.

Subjects were asked to record the intensity of pain on a VAS (visual analogue scale) graded from 0 to 10 with 0 being no pain at all and 10 being the worst pain possible. The period of analgesia due to the nerve block was considered as the period between the time of onset to the time when the pain was marked as mild-moderate.

The pain was estimated by Visual Analogue Scale (VAS) scores and number of analgesics consumed, recorded every 24 h for 1 week.

The swelling was estimated by recording facial measurements 24 h (postoperative day 1 – POD1), 48 h (postoperative day 2 – POD2) and 1 week (post-operative day 7 – POD7) following the procedure.

Trismus was estimated by recording maximal interincisal distance 24 h, 48 h and 1 week following the procedure. Subjects were recalled for the procedure on the other side after 4 weeks, following the same protocol.

Data was analysed using IBM SPSS Statistics for Windows, Version 25.0. Armonk, NY: IBM Corp.

Quantitative variables like onset and duration of anaesthesia; facial swelling, pain and mouth opening were reported in terms of mean and standard deviation to compare test and control group.

To compare the onset and duration of anaesthesia; facial swelling, pain and mouth opening between the test and control group independent *t*-test was applied.

Repeated measures of ANOVA were applied to compare the swelling and mouth opening pre-operatively, 24 h, 48 h and 1 week after the procedure between the test and control group. *p*-value of <0.05 was considered significant.

The normality of the data was tested and the outcome variables were found to follow normal distribution, hence the *t*-tests were used in the statistical analyses.

Consort guidelines (11) were used to report the methodology. This trial was registered in CTRI (CTRI registration number – CTRI/2021/08/035560).

## Results

**Figure 1:**
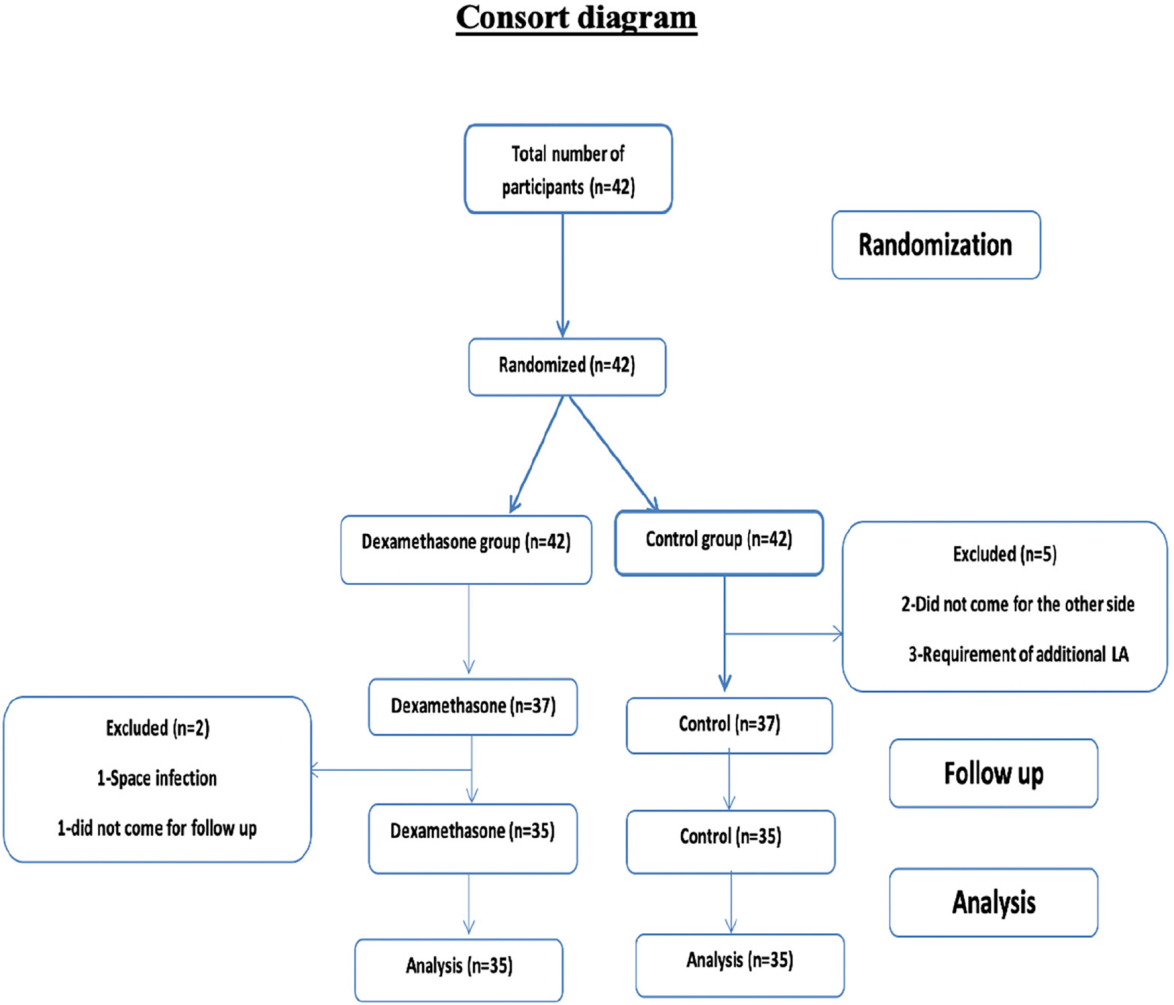
Consort diagram showing patient recruitment and randomisation.

Patient recruitment and randomisation were done as shown in Figure 1.

The onset and duration of anaesthesia observed in the test and control group are shown in Table 1.

**Table 1:** Onset and duration of anaesthesia observed in test group and control group.

Parameter	Test group		Control group		*p*-value
	Mean	SD	Mean	SD	
Onset (seconds)	118.7	34.7	187.7	52.5	<0.001
Duration (minutes)	240.3	44.3	111.9	24.3	<0.001
Independent *t*-test					

**Figure 2:**
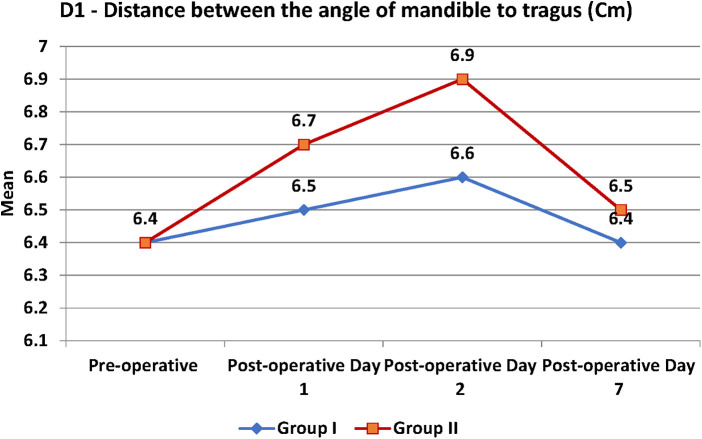
D1- distance between angle of mandible and Tragus of ear.

**Figure 3:**
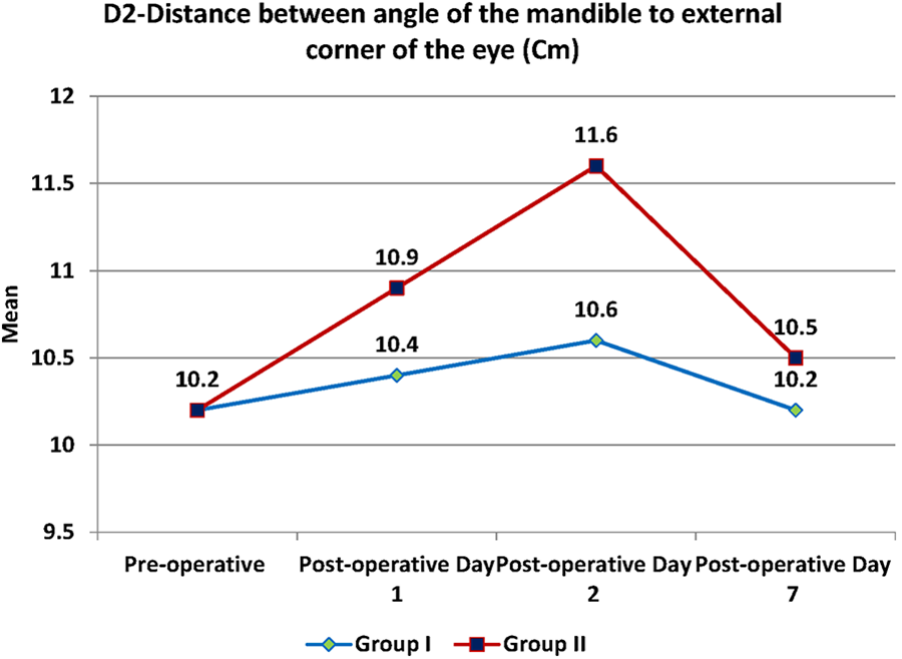
D2- distance between angle of mandible and external canthus of the eye.

**Figure 4:**
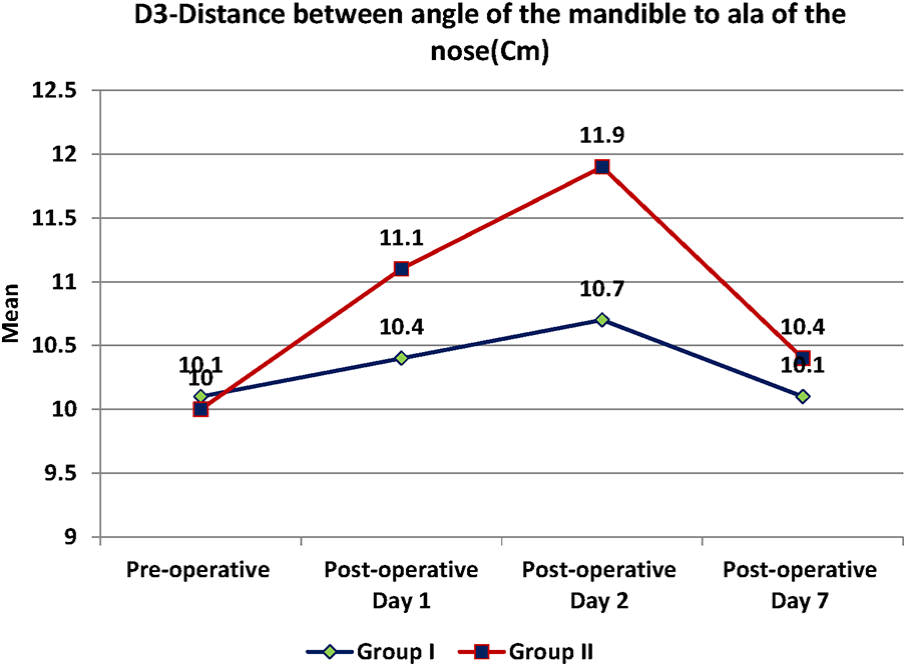
D3- distance between angle of mandible to ala of the nose.

**Figure 5:**
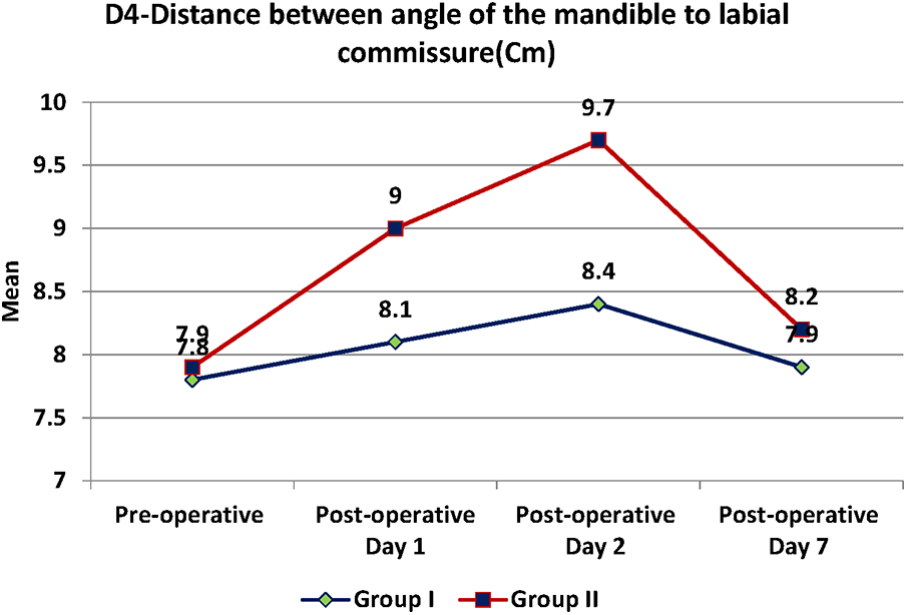
D4- distance between angle of mandible to corner of mouth.

Swelling measured in terms of distances from fixed landmarks on the face (D1, D2, D3, D4, D5) (Figures 2–5) was significantly (*p*-value <0.001)less in the test as compared to the control group (Tables 2–6).

**Table 2:** D1-Distance between angle of mandible to tragus.

D1	Test		Control		*p*-value
	Mean	SD	Mean	SD	
Pre-operative (Cm)	6.4	0.9	6.4	0.9	0.999
Post-operative Day 1 (Cm)	6.5	0.9	6.7	1	0.408
Post-operative Day 2 (Cm)	6.6	1	6.9	1.1	0.135
Post-operative Day 7 (Cm)	6.4	0.9	6.5	1	0.766
Between subjects (groups)					0.500
Within subjects (over time)					<0.001

The distance between the the angle of mandible to tragus was 6.4 cm at the start between the groups. Then it was 6.5 cm on the post-operative day (POD) 1, 6.6 cm on POD 2 and 6.4 cm on POD 7 in group I. In group II, it was 6.7 cm on POD1, 6.9 cm on POD 2 and 6.5 cm on POD 7 (Table 2). There was no significant difference in the distance between the the angle of mandible to tragus at different time points between the groups with the *p*-value of >0.05. There was a significant difference observed with in the subjects over the time with the *p*-value of <0.001.

**Table 3:** D2-Distance between angle of mandible to external canthus of the eye.

D2	Test		Control		*p*-value
	Mean	SD	Mean	SD	
Pre-operative (Cm)	10.2	1.1	10.2	1.1	0.933
Post-operative Day 1 (Cm)	10.4	1.2	10.9	1.2	0.120
Post-operative Day 2 (Cm)	10.6	1.2	11.6	1.4	0.004
Post-operative Day 7 (Cm)	10.2	1.1	10.5	1.2	0.362
Between subjects (groups)					0.149
Within subjects (over time)					<0.001

The distance between the angle of mandible and external corner of the eye was 10.2 cm at the start between the groups. Then it was 10.4 cm on the post-operative day (POD) 1, 10.6 cm on POD 2 and 10.2 cm on POD 7 in test group. In control group it was 10.9 cm on POD1, 11.6 cm on POD 2 and 10.5 cm on POD 7 (Table 3). On POD 2, there was a significant difference of one centimeter in the size of the swelling between the groups with the *p*-value of 0.004. Test group had comparatively better performance. Apart from POD 2, there was no significant difference in the swelling size at different time points between the groups with the *p*-value of >0.05. There was a significant difference in size of the swelling within the subjects over the time with the *p*-value of <0.001.

**Table 4:** D3-distance between angle of mandible to ala of the nose.

D3	Test		Control		*p*-value
	Mean	SD	Mean	SD	
Pre-operative (Cm)	10.1	1	10	1	0.952
Post-operative Day 1 (Cm)	10.4	0.9	11.1	1	0.003
Post-operative Day 2 (Cm)	10.7	0.9	11.9	0.9	<0.001
Post-operative Day 7 (Cm)	10.1	1	10.4	1	0.247
Between subjects (groups)					0.016
Within subjects (over time)					<0.001

The distance between the angle of mandible to ala of the nose was 10.1 cm in group I and 10 cm in group II at the start between the groups. Then it was 10.4 cm on the post-operative day (POD) 1, 10.7 cm on POD 2 and 10.1 cm on POD 7 in test group. In control group, it was 11.1 cm on POD1, 11.9 cm on POD 2 and 10.4 cm on POD 7 (Table 4). There was a significant difference of 0.7 and 1.2 cm in swelling size in the 1st and 2nd POD between the groups with the *p*-value of 0.003 and <0.001. Test group had comparatively better performance in terms of swelling size. Significant difference in size of the swelling within the subjects and between the groups over the time and was observed with the *p*-value of <0.001 and 0.016.

**Table 5:** D4-Distance between angle of mandible to corner of mouth.

D4	Test		Control		*p*-value
	Mean	SD	Mean	SD	
Pre-operative (Cm)	7.8	0.9	7.9	0.9	0.939
Post-operative Day 1 (Cm)	8.1	0.9	9	1	<0.001
Post-operative Day 2 (Cm)	8.4	1	9.7	1.1	<0.001
Post-operative Day 7 (Cm)	7.9	0.9	8.2	0.9	0.184
Between subjects (groups)					0.007
Within subjects (over time)					<0.001

The distance between the angle of mandible to labial commissure was 7.8 cm in group I and 7.9 cm in group II at the start between the groups. Then it was 8.1 cm on the post-operative day (POD) 1, 8.4 cm on POD 2 and 7.9 cm on POD 7 in group I. In group II, it was 9.0 cm on POD 1, 9.7 cm on POD 2 and 8.2 cm on POD 7 (Table 5). There was a significant difference of 0.9 and 1.3 cm in swelling size in the 1st and 2nd POD between the groups with the *p*-value <0.001. The size of the swelling in the post-operative period was minimal in test group as compared to control group. Significant difference in size of the swelling within the subjects and between the groups over the time and was observed with the *p*-value of <0.001 and 0.007.

**Table 6:** D5-Distance between angle of mandible soft tissue pogonion.

D5	Test		Control		*p*-value
	Mean	SD	Mean	SD	
Pre-operative (Cm)	11.4	0.9	11.4	0.9	0.979
Post-operative Day 1 (Cm)	11.6	0.9	11.9	1.1	0.176
Post-operative Day 2 (Cm)	11.7	0.9	12.2	1	0.046
Post-operative Day 7 (Cm)	11.4	0.9	11.6	1	0.524
Between subjects (groups)					0.290
Within subjects (over time)					<0.001

The distance between the angle of mandible to soft tissue pogonion was 11.4 cm at the start between the groups. Then it was 11.6 cm on the post-operative day (POD) 1, 11.7 cm on POD 2 and 11.4 cm on POD 7 in group I. In group II, it was 11.9 cm on POD 1, 12.2 cm on POD 2 and 11.6 cm on POD 7 (Table 6). There was a significant difference of 0.5 cm in swelling size on the 2nd POD between the groups with the *p*-value 0.046. Significant difference in size of the swelling within the subjects over the time and was observed with the *p*-value of <0.001.

**Figure 6:**
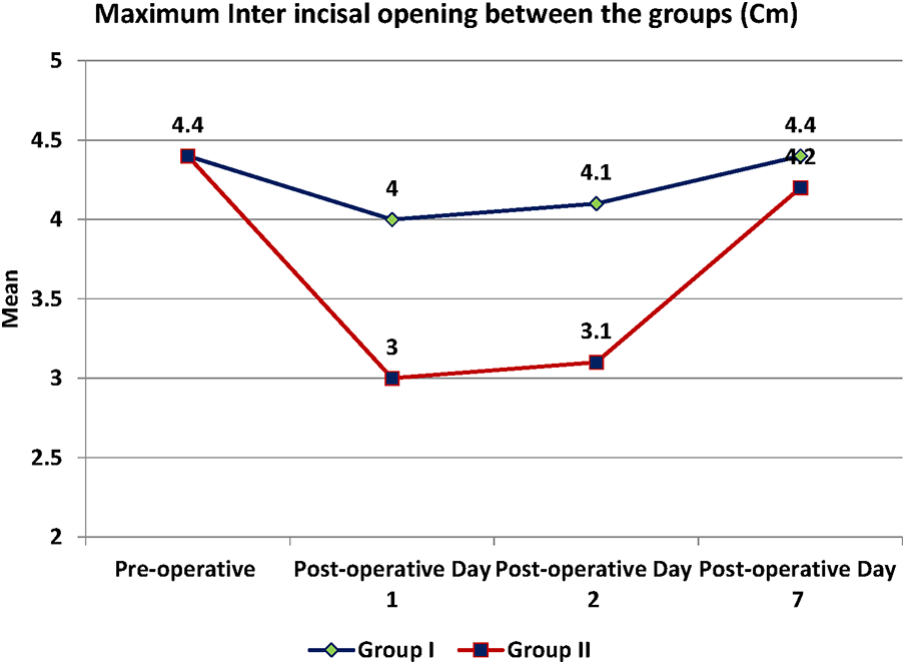
Mouth opening as maximum interincisal distance.

Trismus was quantified by mouth opening measured as the maximum inter-incisal distance (MID) and was significantly more in the test as compared to the control group with a *p*-value <0.001 (Figure 6). MID was 4 cm on the POD 1, 4.1 cm on the POD 2 and 4.4 cm on the 7th day of the post-operative period in the test group.

**Figure 7:**
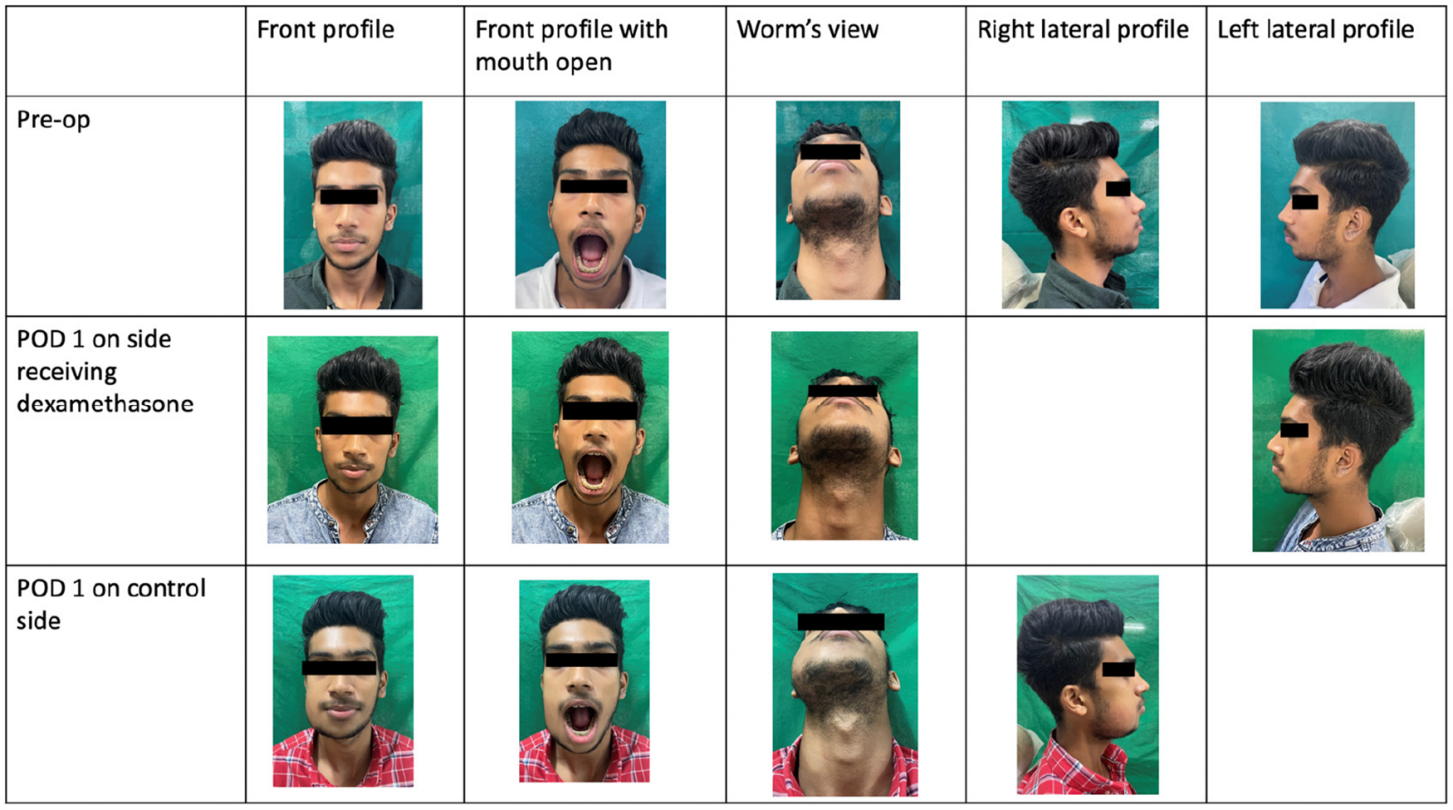
Pre-operative and post-operative clinical images.

In the control group, MID was reduced to 3 cm on POD 1, then 3.1 cm on POD 2 and remained at 4.2 cm on the 7th postoperative day (Table 7). The pre-operative and post-operative clinical images of a subject are presented in Figure 7.

**Table 7:** Mouth opening as maximum interincisal distance.

Mouth opening (Maximum Inter incisal distance)	Test		Control		*p*-value
	Mean	SD	Mean	SD	
Pre-operative (Cm)	4.4	0.4	4.4	0.4	0.976
Post-operative Day 1 (Cm)	4	0.4	3	0.4	<0.001
Post-operative Day 2 (Cm)	4.1	0.4	3.1	0.4	<0.001
Post-operative Day 7 (Cm)	4.4	0.4	4.2	0.4	0.013
Between subjects (groups)					<0.001
Within subjects (over time)					<0.001

The mouth opening (Maximum Inter-incisal distance) was 4.4 cm in the pre-operative period in both the groups. In group I the distance was 4 cm on the POD 1, 4.1 cm on POD 2 and 4.4 cm on 7th day of post-operative period. In group II, the distance was reduced to 3 cm on the POD 2, then 3.1 cm on POD 2 and reached 4.2 cm on the 7th post-operative day (Table 7). There was a significant difference in mouth opening between the groups at 1st, 2nd and 7th post-operative days with the *p*-value of <0.05. Participants in the test group had a better mouth opening in the post-operative days compared to the control.

There was a significant difference observed between the groups and within the subjects over the time with the *p*-value of <0.001.

**Figure 8:**
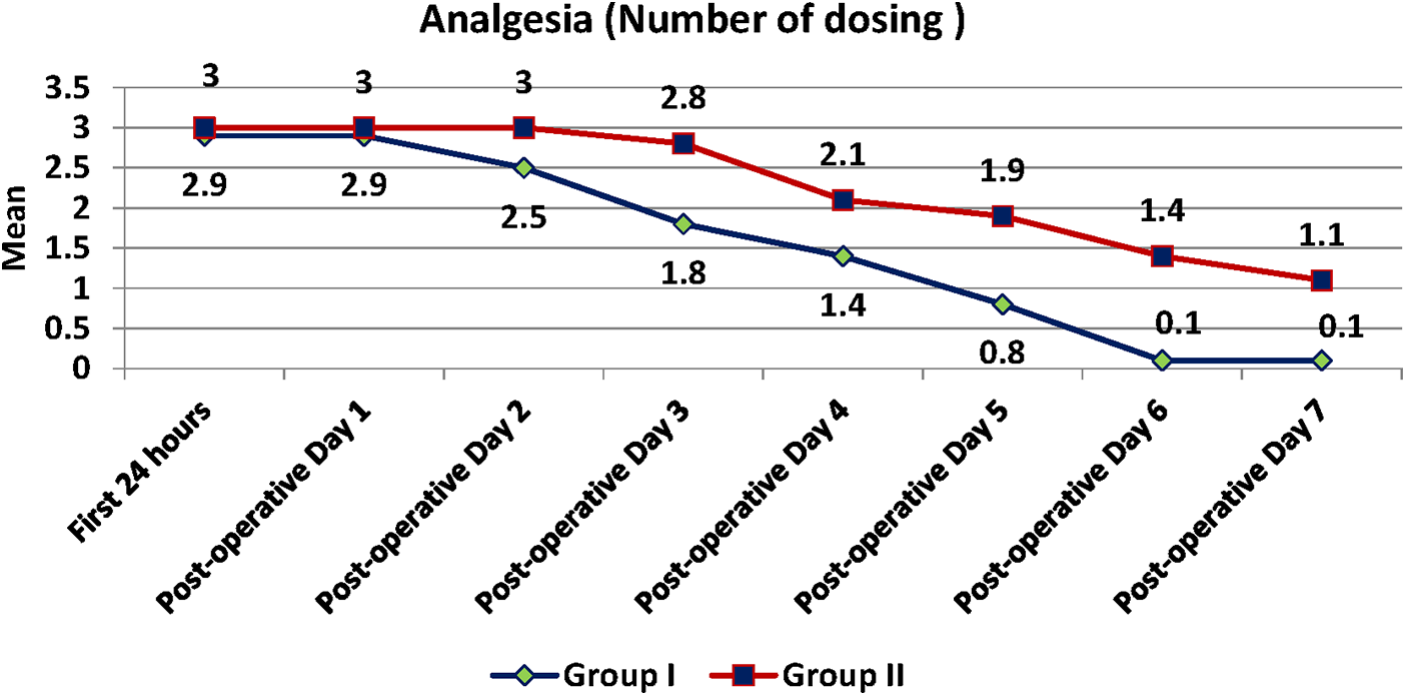
Number of dosing of analgesics between test and control group recorded every 24 h for the first seven days postoperatively.

**Figure 9:**
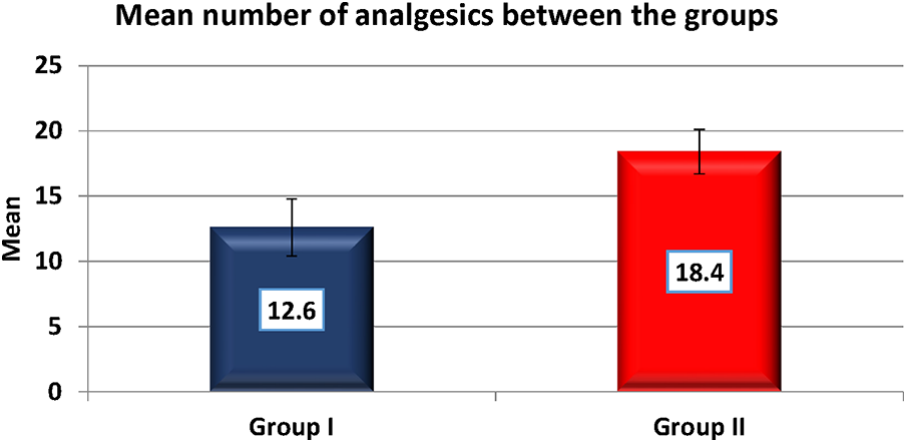
Mean number of total analgesics consumed by test and control group.

Pain was measured using VAS scores (Table 8) and based on number of analgesics consumed every 24 h over the first 7 days (Figure 8). The mean number of analgesics consumed between the groups is shown in (Figure 9) Pain in the test group was significantly less than the control in both parameters with a *p*-value of <0.001.

**Table 8:** Pain scores using visual analogue scale.

Pain scores (VAS)	Test				*p*-value
	Mean	SD	Mean	SD	
First 24 h	4.9	0.7	7.6	0.5	<0.001
Post-operative Day 1	4.5	0.7	7.5	0.6	<0.001
Post-operative Day 2	3.9	0.6	6.6	0.9	<0.001
Post-operative Day 3	2.9	0.8	5.9	0.9	<0.001
Post-operative Day 4	1.9	0.8	4.6	1.2	<0.001
Post-operative Day 5	0.7	0.6	3.3	1.2	<0.001
Post-operative Day 6	0.1	0.3	2.3	1.1	<0.001
Post-operative Day 7	0	0.2	1.5	1.1	<0.001
Between subjects (groups)					<0.001
Within subjects (over time)					<0.001

The timing of consumption of the first analgesic was delayed in the test group as compared to the control by 2 h which coincides with the duration of anaesthesia perceived by the patient by feeling mild-moderate pain. This was statistically significant with a *p*-value of <0.001. There is also a statistically significant difference in the total number of analgesics consumed over 7 days between the groups (Test is 12.6 and Control is 18.4) with a *p*-value of <0.001.

## Discussion

Dexamethasone is a potent anti-inflammatory drug used to offset pain associated with surgical procedures. Different routes of dexamethasone administration have been reported in the literature, and have their pros and cons, but few reports have been published on local anaesthetic agents combined with steroids to offset the post-operative sequelae.

Third-molar surgeries often result in pain, swelling, and trismus, and can deteriorate quality of life, especially during the first 3 days (37). While a normal tooth extraction itself is not a very pleasant experience, surgical extraction of an impacted mandibular third molar is technique-sensitive, violates the integrity of hard and soft tissues, and has a high risk of infection considering its anatomic location juxtaposed to primary spaces of the head and neck (38–40).

The severity of these postoperative sequelae depends on the handling of soft tissues during the intraoperative period, the amount of bone removal and the duration of the surgical procedure.

Maximum pain following surgical extraction of the mandibular third molar is experienced between 3 and 5 h postoperatively (41, 42).

If adequate pain control is not provided during this critical period, mechanical sensitisation of the nerve takes place resulting in a state of hyperalgesia (43). This necessitates the need for pre-emptive analgesia or a higher dose of analgesic. considering the short duration of action of lignocaine. The addition of Bupivacaine can offset the pain and minimise the use of analgesics however its use is limited considering the risk of cardiotoxicity (44).

Therefore, a two-pronged approach of increasing the duration of anaesthesia and reducing post-operative inflammation is vital to minimise the discomfort following surgical extraction of impacted mandibular third molars.

In the present study, we compared the effect of the addition of 8 mg Dexamethasone to 2% Lignocaine with adrenaline against a control group receiving 2% Lignocaine with adrenaline mixed with sterile water for injection to be used as a nerve block. The onset and duration of anaesthesia following the administration of the above mixtures as a nerve block was recorded. In addition, postoperative sequelae including swelling, trismus and pain were also evaluated.

Paracetamol was chosen as the rescue medication, a moderately efficient analgesic with close to no anti-inflammatory effect since it is a weak COX inhibitor (45).

The current study reveals the addition of dexamethasone as an adjunct to lignocaine in the nerve block reduces the time of onset and significantly prolongs the duration of anaesthesia assisting the patient to endure the maximum pain seen in the first 3–4 h.

Further, a low pain score both in the first 24 h and for the next 7 days with less consumption of analgesics was recorded in the test group.

The mechanism for prolonged anaesthesia by glucocorticoids can be attributed to its ability to inhibit potassium channel-mediated discharge of nociceptive C fibres by binding to glucocorticoid receptors on ion channels (46). While this effect cannot produce a state of anaesthesia in itself, it can prolong the duration of anaesthesia, when given perineurally along with an anaesthetic agent by retaining the nerve membrane in an extended hyperpolarised state (47).

The findings of our study in terms of onset and duration are in tandem with the anaesthesia studies, where a significant reduction in latency and prolongation of anaesthesia were observed when dexamethasone was given perineurally along with the Local anaesthetic bupivacaine (48, 49).

A study by Movafegh et al. on the effect of dexamethasone added to lidocaine showed that the duration of sensory blockade of axillary brachial plexus block was significantly longer by 144 min with a *p*-value of <0.001 (50).

Corticosteroids induce the synthesis of endogenous proteins, which block the activation of phospholipase A2, further inhibiting the release of arachidonic acid. This inhibits the release of prostaglandins, leukotrienes and other mediators responsible for inflammation and pain. Unlike NSAIDs, Corticosteroids exert their anti-inflammatory and analgesic action at the initial step of the cascade and are more effective when given before the procedure (51).

Dexamethasone is also known to cause mild to moderate vasoconstriction which in turn holds the local anaesthetic agent perineurally for a longer duration and hence anaesthesia lasts longer (52). Along with this, significantly reduced swelling and trismus can also be attributed to the co-administration of dexamethasone with lignocaine, owing to the well-known anti-inflammatory effects of corticosteroids. The mechanism of action of early onset due to dexamethasone is not clear yet, though there is definite clinical evidence in support. Further research in this aspect is required to understand this phenomenon.

The use of corticosteroids has gained traction in the field of oral and maxillofacial surgery however, the most variable aspect of the utility of corticosteroids is the most appropriate route of administration. Different administration routes systemic or local have their merits and drawbacks. Intramuscular, Intravenous, Oral, Submucosal and Endoalveolar powder are the various routes reported in the literature (53). The twin mixture of local anaesthesia and steroids used in this study has a quadruple benefit all of which can be attained with a single prick.

Drug absorption largely depends on the vascularity in the area of administration. The Pterygomandibular space is a robust vascular zone, filled with loose areolar tissue space, and lacks dense fibrous elements, all of which permit swift diffusion, and absorption of local anaesthetic with minimal needle deflection (54). It is the zone of insertion during the administration of an inferior alveolar nerve block.

The mandible has a cortical rim with a bulky medullary core, and hematopoietic marrow that persist in the ramus and condyle, even at the age of 25 years (55). This type of bone marrow presents a network of capillaries and veins with a disjointed endothelium that permits fluids and other substances in the surrounding stroma to be readily swapped with the blood current (1, 55). This unique feature of the mandible can also enable faster diffusion of anaesthetic when injected intra-osseously.

Local administration of steroids seems to be more beneficial as they act directly on the eicosanoids that are released from the tissues during injury and hence prevent inflammatory processes (56, 57). Eicosanoids are a class of molecules, derivative from 20-carbon (“eicosa” is Greek for 20) polyunsaturated fatty acids, most commonly arachidonic acid. The eicosanoids are the key mediators and regulators of inflammation and immunity and include prostaglandins, thromboxanes, leukotrienes, and lipoxins (57–59).

Steroids are beneficial in the post-operative period, but their utility in oral and maxillofacial surgeries is yet to be a part of the protocol. We recommend the steroid local anaesthetic mixture to minimise the inevitable post-operative sequelae and as observed based on our study, the formulated mixture did not cause any adverse effect.

The anti-inflammatory action of dexamethasone is 20–30 times higher than cortisol, and a t1/2 of 36–54 h makes it the drug of choice as a single dose in the management of collateral effects associated with surgical removal of third molars (60, 61).

Our study also aligns with the results of the Shivanagi et al. study that concluded Intraoperative and postoperative comfort in the test group were higher than those for control groups, thereby establishing the clinical efficacy twin mixture for use in surgical extraction of mandibular third molars. The only difference was the local anaesthetic agent comprising of Bupivacaine and Ropivacaine (62).

Limited studies are done on the effect of the addition of dexamethasone to LA to hasten its onset and duration. Further large-scale studies are warranted to validate the efficacy of this mixture of local anaesthetic and steroids, measure the plasma level of steroids with the different routes of administration and its utility in providing adequate depth and duration of anaesthesia and minimising or eliminating the need for analgesics. The dexamethasone dosage also needs to be standardised in the literature, when evaluating postoperative parameters.

Clinically, dexamethonized Lignocaine has shown promising results in this study, reducing the postoperative sequel of pain, trismus and swelling in patients undergoing surgical removal of impacted third molars. However, the long-term chemical stability of these solutions is beyond the scope of this study. Further studies on the synthesis, storage, latent effect and shelf life of these solutions are necessary for optimum usage in everyday practice.

The positives of the study were a standardized study design with 100% follow-up. The procedure was executed by a single operator thereby negating operator bias. A possible interpersonal difference in pain perception was ruled out due to the split-mouth design.

Postoperative oedema varies according to local factors such as the position of the impacted teeth, method of bone removal, haemostasis, over-suturing of the wound, traumatic handling of soft and hard tissues and systemic factors such as age, bleeding tendency, nutrition, use of drugs, or presence of diabetes (63). The limitations of our study include, there are several contributing factors increasing the risk of pain, oedema and trismus, and inflammatory process, and it is difficult to validate if dexamethasone can influence every contributing factor. Split mouth design was used to overcome systemic factors and local factors contributing to the post-operative sequelae of third molar surgery. Also, the inevitable time duration considering the volume of injection administered compared to the conventional 2 ml can be a source of discomfort, especially in an apprehensive patient. Further studies with larger samples and measurement of plasma levels of dexamethasone following its administration are warranted.

## Conclusion

Our study concludes that the addition of dexamethasone to lignocaine with adrenaline reduces the latency and prolongs the duration of local anaesthetic action enabling the individual to endure the period of maximum pain. Pain is attenuated as evidenced by the low pain score in the first 24 h, ensuing 7 days and also lesser number of overall analgesics consumed by patients receiving this intervention. In addition the local anaesthetic steroid mixture also minimises secondary outcomes like swelling and trismus.

## Data Availability

The original contributions presented in the study are included in the article/supplementary material, further inquiries can be directed to the corresponding author.
